# Accuracy of telephone triage for predicting adverse outcomes in suspected COVID-19: An observational cohort study linking NHS 111 telephone triage, primary and secondary healthcare and mortality records.

**DOI:** 10.23889/ijpds.v7i3.1777

**Published:** 2022-08-25

**Authors:** Carl Marincowitz, Tony Stone, Peter Bath, Richard Campbell, Janette Turner, Richard Pilbery, Benjamin Thomas, Laura Sutton, Fiona Bell, Richard Pilbery, Katie Biggs, Frank Hopfgartner, Madina Hussein, Suvodeep Mazumdar, Jennifer Petrie, Steve Goodacre

**Affiliations:** 1 School of Health and Related Research, University of Sheffield; 2 Information School, University of Sheffield; 3 Yorkshire Ambulance Service NHS Trust

## Objectives

To assess accuracy of telephone triage in identifying need for emergency care amongst those with suspected COVID-19 infection and identify factors which affect triage accuracy.

## Approach

An observational cohort study of adults who contacted the NHS 111 telephone triage service provided by Yorkshire Ambulance Service between March and June 2020 with symptoms indicating possible COVID-19 infection. Patient-level data encompassing triage call, primary care, hospital care and death registration records relating to 40,261 adults were linked.

The accuracy of triage outcome (self-care/non-urgent assessment versus ambulance/urgent assessment) was assessed for death or organ support 30 days from first contact. Multivariable logistic regression was used to identify factors associated with risk of false negative or false positive triage.

## Results

Callers had a 3% (1,200/40,261) risk of serious adverse outcomes. Telephone triage recommended self-care or non-urgent assessment for 60% (24,335/40,261), with a 1.3% (310/24,335) risk of adverse outcomes 30 days from first contact. Telephone triage had 74.2% sensitivity (95% CI: 71.6 to 76.6%) and 61.5% specificity (61% to 62%) for the primary outcome. Analysis suggested respiratory comorbidities may be over-appreciated and diabetes under-appreciated as predictors of deterioration. Repeat contact with triage service appears to be an important under-recognised predictor of deterioration.

## Conclusion

Patients advised to self-care or receive non-urgent clinical assessment had a small but non-negligible risk of serious clinical deterioration. Repeat contact with telephone services needs recognition as an important predictor of subsequent adverse outcomes.

